# Phenotypic Genetic Analysis of Fruit Branch Angle in Upland Cotton

**DOI:** 10.3390/plants14101512

**Published:** 2025-05-18

**Authors:** Yanping Tan, Yilei Long, Yinan Yang, Yin Wang, Shen Jin, Xiantao Ai

**Affiliations:** 1 College of Life Science and Technology, Xinjiang University, Urumqi 830017, China; 107552200991@stu.xju.edu.cn (Y.T.); longyilei@stu.xju.edu.cn (Y.L.); 107552401014@stu.xju.edu.cn (Y.Y.); 107552301030@stu.xju.edu.cn (Y.W.); 107552400997@stu.xju.edu.cn (S.J.); 2 College of Smart Agriculture (Research Institute), Xinjiang University, Urumqi 830017, China

**Keywords:** upland cotton, fruit branch angle, different regions, different periods

## Abstract

This study aims to reveal the genetic variation of fruit branch angle (FBA) in upland cotton, thereby providing a scientific basis and practical guidance for cotton architecture breeding. We explored the genetic variation pattern of FBA in 300 upland cottons from different regions and different periods, respectively. Cluster analysis and principal component analysis were used to comprehensively evaluate the plant architecture traits and yield traits in 300 upland cottons. The results demonstrated that the range of variation of FBA in cotton was 43.59–69.32°, the coefficient of variation ranged from 6.06% to 7.42%, and the broad-sense heritability was 75.50%. The order of FBA in different regions was as follows: Foreign Germplasm (FG; 56.77°) > Yellow River Region (YRR; 56.24°) > Yangtze River Region (YZRR; 56.16°) > Liaoning Special Maturing Region (LSMR; 55.35°) > Northwest Inland Region (NIR; 55.25), which is rich in genetic diversity. FBA in cotton in different periods had obvious differences. FBA was the largest before 1960, and as the period progressed, FBA showed an overall fluctuating decrease, whose coefficient of variation and genetic diversity index tended to increase. In this study, it was found that when the range of FBA was 50.46–55.31°, cotton had the best overall performance, with compact architecture, fewer empty fruit branches, more bells, and higher yield, which can be further developed and utilized as an excellent cotton germplasm resource.

## 1. Introduction

As a natural fiber product, cotton plays an irreplaceable role in China’s economic growth and social development, and ensuring the stable and sustainable development of the cotton industry is vital to the country’s long-term development. ‘Machine-harvested cotton’ is the trend of cotton industry development in Xinjiang. In order to meet the demand for high-density planting and mechanical harvesting, Xinjiang cotton needs to build a reasonable plant architecture, thereby improving planting density and mechanical harvesting efficiency to achieve higher quality and greater efficiency.

Plant architecture refers to the overall morphological traits and spatial organization of crop developed during the growth process, including the plant height of the crop, the number of branches, branching angle, and leaf arrangement [1]. Ideal architecture improves crop leaf area index, population photosynthetic efficiency, and yield, indicating that strain type is closely related to crop production and breeding [2]. In the architecture traits of cotton, fruit branch angle (FBA) refers to the angle between the main stem and the fruit branches of cotton. This trait is a critical determinant of cotton plant architecture, influencing the spatial distribution of reproductive structures (flowers and bolls) and contributing to overall plant compactness [3]. Planting density [4], chemical regulation [5,6], water and fertilizer management [7,8], and photosynthetic efficiency [9] all affect the final yield of many crops by altering branching angles. Many studies have shown that cotton FBA are typical quantitative traits that are co-regulated by genotype and environmental factors [10,11,12]. At the genotypic level, genes such as *LAZY1* [13,14], *TAC1* [15], *TAC3* [16], *TAC4* [17], and *D2* [16] have been identified as the main effector genes controlling branch angle formation. Among environmental factors, gravity and light play a major role in branch angle formation [18]. Gravity plays a crucial role in dynamically modifying plant growth direction during their development. This process can be categorized into four key stages: (1) gravity signal perception, (2) signal transduction, (3) formation of asymmetrically distributed auxin (IAA), and (4) curvature growth of gravitropic organs [19]. Light is able to regulate the branching angle of the crop for optimal light energy capture efficiency [20]. Other plant traits also play a key role in cotton yield formation. Nan et al. [21] suggested that the morphological characteristics of high-yielding cotton are short plants, short fruit branches, small and thick leaves, and large bells. Ma et al. [22] used 128 early maturity cotton plants from Xinjiang as experimental materials to study the correlation between plant architecture and yield, finding that plant height, number of bells, and the first fruit branch node had direct and significant impacts on yield, whereas the number of fruit branch tiers exerted an indirect yet significant influence. Zhu et al. [23] divided the test material into four types of plant architectures according to the cotton fruit branch angle and main stem pitch, explored its effect on yield, and found that a small fruit branch angle, large main stem pitch, distinctive leaf level, large leaf angle, and small number of fruit nodes were conducive to yield formation.

With the wide application of cotton mechanical harvesting technology in Xinjiang, the requirements for cotton plant architecture traits adapted to mechanical harvesting are also increasing. Thus, researchers must intensify efforts in cotton plant architecture breeding to develop improved varieties optimized for mechanical harvesting, thereby enhancing both yield and fiber quality. In this study, we used 300 upland cottons as experimental materials, focusing on analyzing the variation in FBA in different regions and different periods. Through cluster and principal component analyses, the optimal FBA group was investigated by combining architecture traits and yield traits, aiming to provide theoretical guidance for the breeding of cotton architecture.

## 2. Results

### 2.1. Phenotypic Analyses of Fruit Branch Angle Traits in Cotton

**Figure 1:**
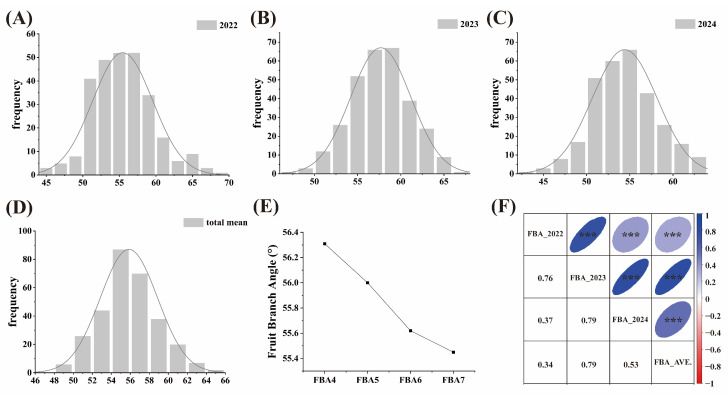
Phenotypic analysis of FBA in cotton. (**A**–**D**) Histogram of frequency distribution of cotton fruit branch angle in 2022, 2023, 2024, and total mean; (**E**) FBA analysis of 4th, 5th, 6th, and 7th-stage fruit branches in cotton; (**F**) correlation analysis of FBA in cotton. *** Correlations are significant at the 0.001 level.

We performed descriptive statistical analyses of FBA in 300 upland cottons. As shown in Table 1, the average of FBA in 2022 was 55.46°, the Coefficient of Variation (CV) was 7.42%, and the range of variation was between 44.57 and 69.32°; the average of FBA in 2023 was 57.68°, the CV was 6.06%, and the range of variation was between 46.23 and 66.07°; the average of FBA in 2024 was 54.41°, the CV was 6.93%, and the range was 43.59–63.54°; the average of the total means was 55.84°, the CV was 5.31%, and the range was 47.60–65.16°. The CV of FBA ranged from 6.06% to 7.42% during the three years, which was practically unchanged, demonstrating relatively consistent variation in cotton FBA. Analysis of three-year data revealed that FBA in cotton exhibited a broad-sense heritability (*H*^2^) of 75.50%, demonstrating that FBA in cotton is mainly influenced by genetic factors and relatively less by the environment. According to the normal distribution analysis, the absolute values of skewness and kurtosis of FBA were both less than 0.6, suggesting that the distribution of FBA in cotton followed an approximately normal pattern (Table 1). This conclusion was further supported by the frequency distribution histogram (Figure 1A−D). The FBA in 2024 was significantly smaller than the total mean, whereas the FBA in 2023 was significantly larger than the total mean, which may be related to climate change in recent years (Table 1). We analyzed the FBA of the 4th, 5th, 6th, and 7th-stage fruit branches in cotton, and we found that the FBA became smaller as the stage number of fruit branches increased, which was consistent with the phenomenon observed in the field (Figure 1E). Correlation analysis showed significant positive correlations between 2022, 2023, 2024, and the total mean; among them, the FBA correlation between 2023, 2024, and the total mean was the highest at 0.79, and the FBA correlation between 2022 and the total mean was the lowest at 0.34 (Figure 1F).

### 2.2. Variation Analysis of FBA in Cotton in the Different Regions

The Yellow River Region (YRR), Yangtze River Region (YZRR), Liaoning Special Maturing Region (LSMR), and Northwest Inland Region (NIR) are the four major cotton regions in China, with significant differences in natural environment, climate characteristics, and ecosystems. Therefore, key aspects such as variety selection strategies, field management measures, and pest and disease prevention and control systems used in different cotton regions also vary from place to place, resulting in their own unique production models [24]. Therefore, we divided the 300 upland cottons into five groups according to the breeding regions, including YRR, YZRR, LSMR, NIR, and Foreign Germplasm (FG). According to Table 2, the 300 upland cottons were widely distributed in the different regions. Specifically, the YRR included 99 accessions, accounting for 33.0% of the total population; the LSMR included 43 accessions, representing 14.3% of the total population; the NIR included 89 accessions, representing 29.7% of the total population; the YZRR included 42 accessions, representing 14.0% of the total population; and the FG included 27 accessions, accounting for 9.0% of the total population. Significant regional variations were observed in the FBA of cotton, with the four major cotton-producing regions in China exhibiting smaller FBA values compared to the FG (56.77°). The FBA of cotton from the four major cotton-producing regions in China was ranked in the following order: YRR (56.24°) > YZRR (56.16°) > LSMR (55.35°) > NIR (55.25°). It was found that the FBA of the cotton from YRR and YZRR was closer to that of FG, which may be due to the fact that YRR and YZRR had more introductions from foreign countries in the past; the FBA of cotton from LSMR and NIR was significantly lower than that of the above three regions. The FBA was the smallest in NIR, which may be related to its adoption of the ‘short, dense, early’ planting pattern [25,26]. Looking at the genetic diversity index (*H*′), the *H*′ of cotton FBA across five regions ranged from 1.91 to 2.04. The *H*′ of cotton FBA in the YRR was the highest at 2.04, while that in the YZRR was the lowest at 1.91, demonstrating that cotton FBA exhibits considerable genetic diversity across different regions (Table 2). Looking at CV, the FBA in LSMR was the lowest CV (4.15%), while that in NIR was the highest (6.02%) (Table 2). This variation in CV might also be associated with the introduction strategies adopted by the respective regions.

### 2.3. Variation Analysis of FBA in Cotton at Different Periods

The 300 upland cottons are all bred in China or have been introduced from abroad in the past 100 years. We divided them into eight periods (S1–S8), where S1 represents before 1960 and every 10 years thereafter. S1 includes 19 varieties selected before 1960, S2 includes 10 varieties selected in the 1960s, S3 includes 14 varieties selected in the 1970s, S4 includes 15 varieties selected in the 1980s, S5 includes 34 varieties selected in the 1990s, S6 includes 65 varieties selected in the 2000s, S7 includes 70 varieties selected in the 2010s, and S8 includes 20 varieties selected in the 2020s. The FBA in cotton differed considerably in average CV and *H*′ at different periods (Table 3). Before 1960, the FBA in cotton was the highest (57.28°), potentially attributable to the higher frequency of foreign introductions during this period. In contrast, the FBA was the smallest (54.59°) in the 1990s. From S1 to S8, FBA showed an overall fluctuating decrease of 2.07°. Looking at CV, the CV of FBA in the 1960s was the smallest (2.49%), and the CV of FBA in the 2020s was the largest (7.22%). Looking at *H*′, the 1990s is a dividing line, and the *H*′ of cotton FBA before 1990 is overall smaller than that after 1990. In general, it appeared that the CV and *H*′ of FBA in cotton tended to increase with era. We analyzed the FBA of the 4th, 5th, 6th, and 7th-stage fruit branch in cotton separately, and we found that the FBA of each stage fruit branch showed different degrees of variability with era. The FBA of the 4th-stage fruit branch had the smallest variation range and CV (2.65°, 1.51); the FBA of the 7th-stage fruit branch had the largest variation range and CV (3.07°, 2.21). The overall performance showed that the taller the stage of fruit branch, the greater the variability in FBA (Table 4).

### 2.4. Phenotypic Analyses of Architecture Trait and Yield Trait in Cotton

#### 2.4.1. Variance Analysis of Architecture Trait and Yield Trait in Cotton

We measured and analyzed six architecture traits and three yield traits of 300 upland cottons, including Plant Height (PH), Fruit Branch Initiation Node (FBIN), Fruit Branch Initiation Height (FBIH), Number of Fruit Branches (FBN), Number of Effective Fruit Branch (EFBN), Fruit Branch Angle (FBA), Bell Per Plant (BPP), Bell Weight (BW), and Lint Percentage (LP). The results are shown in Table 5. The average PH was 72.60 cm, with a range of variation between 53.38 and 98.47 cm; the average FBIN was 6.05, with a range of variation between 4.52 and 8.31; the average FBIH was 29.24 cm, with a range of variation between 19.52 cm and 42.64 cm; the average FBN was 8.71 units, with a range of variation between 7.29 and 11.17 units; the average EFBN was 5.59 units, with a range of variation between 3.98 and 7.75 units; the average FBA was 55.84°, with a range of variation between 47.60 and 65.16°; the average BPP was 8.50, with a range of variation between 5.65 and 15.45; the average BW was 6.06 g, with a range of variation between 4.79 and 7.95 g; and the average LP was 0.41, with a range of variation between 0.31 and 0.48. We analyzed the CV of six architecture traits and three yield traits, revealing that the CV of the BPP was the largest (17.55%), followed by FBIH (14.70%), EFBN (10.80%), PH (9.52%), FBIN (9.48%), BW (7.34%), FBN (7.25%), and LP (6.37%). Notably, the CV for FBA was the lowest (5.31%), demonstrating its greater stability compared to the other traits.

#### 2.4.2. Correlation Analysis Between Architecture Traits and Yield Traits in Cotton

We conducted simple correlation analyses among six architecture traits and three yield traits in 300 upland cottons (Table 6), which revealed that among the 36 pairs of relationships analyzed, 13 pairs demonstrated highly significant correlations (*p* < 0.01) and 4 pairs exhibited significant correlations (*p* < 0.05). PH was significantly and positively correlated with FBIN (0.335 **), FBIH (0.771 **), EFBN (0.201 **), BPP (0.123 *), and LP (0.183 **); FBIN was highly significantly and positively correlated with FBIH (0.672 **) and significantly and negatively correlated with FBN (−0.434 **) and EFBN (−0.115 *); FBIH was highly significantly negatively correlated with FBN (−0.370 **) and significantly positively correlated with LP (0.182 **); FBN was highly significantly and positively correlated with EFBN (0.329 **), BPP (0.360 **), and highly significantly and negatively correlated with LP (−0.186 **); EFBN was significantly and positively correlated with FBA (0.200 **), BPP (0.675 **), and BW (0.127 *); and FBA was significantly and positively correlated with EFBN (0.200 **) and BW (0.131 *). Among these nine traits, PH had the highest correlation with FBIH, followed by EFBN and BPP, while EFBN had the lowest correlation with BW.

#### 2.4.3. Principal Component Analysis of Architecture Traits and Yield Traits in Cotton

We performed principal component analysis for six architecture traits and three yield traits in three hundred upland cottons (Table 7). The results revealed that the first four principal components had eigenvalues greater than 1, accounting for variance contributions α1, α2, α3, and α4 of 27.263%, 22.257%, 13.156%, and 11.939%, respectively, with a cumulative contribution of 74.616%. This demonstrated that the first four principal components could explain a significant part of the variation in the nine traits. The six architecture traits and three yield traits were set as X_1_, X_2_, X_3_, X_4_, X_5_, X_6_, X_7_, X_8_, and X_9_, respectively. Using the eigenvalues of each index and their corresponding eigenvectors, we derived linear combinations of the four principal components with the original nine indexes as follows:

y_1_ = 0.394X_1_ + 0.510X_2_ + 0.581X_3_ − 0.391X_4_ − 0.159X_5_ − 0.015X_6_ − 0.195X_7_ + 0.002X_8_ + 0.178X_9_, (1)

y_2_ = 0.395X_1_ + 0.042X_2_ + 0.190X_3_ + 0.290X_4_ + 0.595X_5_ + 0.161X_6_ + 0.557X_7_ + 0.015X_8_ + 0.180X_9_, (2)

y_3_ = −0.247X_1_ + 0.090X_2_ − 0.148X_3_ − 0.318X_4_ + 0.075X_5_ + 0.715X_6_ + 0.015X_7_ + 0.495X_8_ + 0.214X_9_, (3)

y_4_ = 0.170X_1_ + 0.131X_2_ + 0.095X_3_ + 0.184X_4_ − 0.068X_5_ − 0.100X_6_ + 0.046X_7_ + 0.655X_8_ − 0.682X_9_, (4)

A principal component composite model was calculated based on the percentage of variance of the four principal components:

Y = 0.273y_1_ + 0.223y_2_ + 0.132y_3_ + 0.119y_4_ (5)

Based on these results, the composite scores for six architecture traits and three yield traits were calculated for the three hundred upland cottons.

#### 2.4.4. Cluster Analysis of Architecture Traits and Yield Traits in Cotton

**Figure 2:**
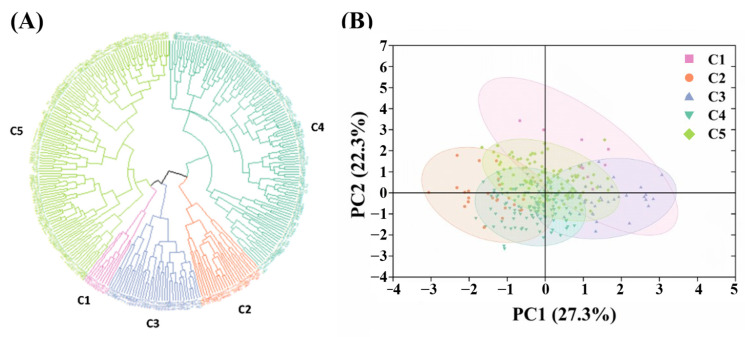
Phenotyping of architecture traits and yield traits in cotton. (**A**) Cluster analysis of architecture traits and yield traits; (**B**) principal component analysis plot.

We performed cluster analysis of 300 upland cottons for architecture traits and yield traits using the Ward.D2 method. Cluster analysis revealed that at a Euclidean distance of 8.5, the materials were classified into five groups, i.e., C1, C2, C3, C4, and C5 (Figure 2A). This classification pattern showed similarity with the principal component analysis plot (Figure 2B). As shown in Table 8, C1 contained nine varieties, accounting for 3.00% of the population, with a composite PCA score of 0.71, of which eight were from the NIR. C2 contained 24 varieties, accounting for 8.00% of the population, with a PCA composite score of −0.82, mainly from the LSMR and NIR. C3 contained 35 varieties, accounting for 11.67% of the population, with a PCA composite score of 0.51, mainly from the NIR and YRR. C4 contained 114 varieties, accounting for 38.00% of the population, with a combined PCA score of −0.34, mainly from the YRR. C5 contained 118 varieties, accounting for 39.33% of the population, with a PCA composite score of 0.29, mainly from the NIR and YRR. Based on the composite model of principal component analysis, we calculated the composite scores for each group. The results showed that the PCA composite scores of C1 and C3 were significantly higher than those of the other groups, with their FBA ranges of 50.46–55.31° and 50.95–61.08°, respectively, which can be further developed and utilized as excellent cotton germplasm resources.

## 3. Discussion

### 3.1. Phenotypic Variability Analysis of FBA in Cotton

Upland cotton is distributed in every major cotton region in China, exhibiting broad phenotypic diversity in FBA traits. Based on long-term production practices, China’s cotton scientists, technological personnel, and the majority of cotton farmers have, according to natural ecological conditions, planting systems, breeding strategies, and other elements in different regions, formed regionally adapted cotton architectures and fruit branch angle characteristics, which have important guiding value for cotton production. In this study, we investigated and analyzed 300 upland cottons for 3 consecutive years for FBA traits. Our results demonstrated that FBA in cotton ranged between 43.59 and 69.32°, with a wide range of variation and a continuous distribution. Ma et al. [3] reported a consistent decreasing trend in FBA from basal to apical positions, identifying middle and upper fruit branches as the most reliable indicators of whole-plant FBA characteristics. Similarly, Li et al. [27] also proved that the FBA of cotton gradually decreased as the number of fruit branch stations increased. Therefore, we chose the FBA of the middle and upper fruit branches in cotton, i.e., the 4th, 5th, 6th, and 7th-stage fruit branches, and the same conclusions were reached. Although plant branch angle is influenced to some extent by factors such as cultivation pattern, chemical regulation, and external growth environment, the genetic characteristics of the plant itself are the main determinants of branching angle [28,29]. Therefore, it is important to elucidate the inheritance regulation of FBA in upland cotton for the theoretical guidance of its architecture breeding. The heritability of FBA in cotton differs according to the genetic population and the environment in which the genetic population is located. Ma et al. [3] used a mixed main gene + polygenic inheritance model to analyze 418 cotton varieties for FBA, and they found that the trait was controlled by two pairs of equal additive main genes, with the heritability of the main genes being 90.22%. Shao et al. [30] used 163 upland cottons as test materials and derived a generalized heritability of 68.70% for the FBA of the cotton in 4 environments over 2 years. Li et al. [8] calculated the broad-sense heritability of FBA in cotton to be 78.98% (measured by AutoCAD) and 67.36% (measured by AM software) after 2 years and at 3 locations. In the present study, we used 300 upland cottons as test materials and calculated the broad-sense heritability of 75.50% of FBA across 3 years and 8 environments. This indicates that FBA traits in cotton are mainly controlled by genetic factors and less influenced by the environment. Therefore, future research on FBA in cotton should be gradually shifted to genome-wide association analysis to explore the main effect genes controlling FBA and to accelerate breeding research on FBA using molecular markers to assist breeding.

### 3.2. Variation Analysis of FBA of Cotton in the Different Regions and Different Periods

The FBA traits of the cotton differed in the different regions. The FBA of the cotton in the NIR was the smallest, which may be related to the ‘short, dense, and early’ planting pattern adopted in the inland northwest region [25,26]. High precipitation and relative humidity are favorable conditions for the development of pests and diseases [31]. The FBA of cotton in the YRR and YZRR was significantly large, which may be related to the humid climate of the two regions. Larger FBA values are conducive to ventilation and light penetration, reduction of diseases, and improvement of photosynthesis efficiency. Notably, the FBA of cotton from the YRR and YZRR as close to that of FG, which may be related to the fact that the YRR and YZRR were the earliest places to introduce foreign germplasm. Looking at CV, the CV of cotton FBA in LSMR was the smallest (4.15%), while the CV of cotton FBA in NIR was the largest (6.02%). This regional disparity might be attributed to distinct introduction strategies: LSMR is the Liao Cotton series, which has a low frequency of introduction and has a more similar architecture, so the FBA varies less and is more stable than the other areas. In contrast, Xinjiang has more introductions from across the world, with a wide range of varieties and abundant architecture, resulting in greater variation in the FBA in cotton. Therefore, in future breeding work, breeders will have to introduce and breed for suitability to different regions to produce new varieties adapted to local environments.

The FBA in cotton differs in different periods. With the passage of era, the FBA in cotton has undergone several fine-tunings, but with an overall fluctuating decline, which may be influenced by the change in cotton planting density in China. Some studies have shown that with the increase of planting density, the FBA angle of cotton will become smaller and the architecture will become more compact [32,33]. The FBA in cotton has gone through multiple stages of adjustment, which are mainly influenced by factors such as variety improvement, technological progress, policy guidance, and mechanization development. Therefore, when breeding new varieties, breeders must take into account all of these influences in a comprehensive and integrated manner in order to breed new high-quality cotton varieties that meet actual needs. Meanwhile, the FBA of the 4th, 5th, 6th, and 7th-stage fruit branches in cotton showed different degrees of variability as the decades went by, among which the FBA of the 4th-stage fruit branch showed the smallest variability (2.65°), while that of the 7th-stage fruit branch showed the largest variability (3.07°), which indicated that the taller the stage number of fruit branches in cotton, the greater the variability of FBA. This may be related to the more complex genetic regulation of the higher fruit branches [34]. In general, as the years progressed, the FBA in cotton exhibited a fluctuating decline, while both CV and *H*′ increased significantly. Notably, the variability characteristics of the higher fruit branches stood out. This indicates that cotton has gradually developed a morphological structure that is more adapted to the current environment over a long period of cultivation and evolution, while individual differences and genetic richness within populations have increased, providing more options for breeding and enhancing its adaptability and resilience.

### 3.3. Optimal Population Analysis of FBA in Cotton

In crops, branching angle is a key agronomic trait that shapes crop morphology, with its size directly influencing planting density, light use efficiency, and final yield [35]. For example, through long-term variety screening and genetic improvement, human beings have gradually bred wild rice with a loose architecture into compact cultivated rice, which has significantly enhanced the adaptability of rice to high-density planting environments and its photosynthetic efficiency, thereby effectively promoting yield growth [16]. For cotton, a smaller FBA facilitates the development of a more compact and optimized canopy architecture. This structural characteristic enhances mechanical harvesting efficiency, improves field ventilation and light penetration within the plant population, and promotes natural leaf abscission during the boll-opening period. These combined effects significantly reduce trash content in machine-harvested cotton while simultaneously improving both yield and fiber quality [36,37]. This has played a significant role in promoting the rational layout of crops, facilitating the operation of machinery and improving the efficiency of agricultural production. In this study, we measured and analyzed six architecture traits and three yield traits, revealing significant to highly significant correlations between plant architecture and yield components. Among them, the correlation between FBA and EFBN was the largest and highly significant, and the positive correlation with BW was also significant. The significant positive correlation between the EFBN and BPP, LP indicated that FBA in cotton can directly or indirectly affect yield. Meanwhile, there was a significant positive correlation between PH, FBN, EFBN, and BPP, as well as a significant positive correlation between PH, FBIH, EFBN, and LP, while there was a highly significant negative correlation between FBN and LP, which was similar to the results of a previous study [38,39].

We carried out cluster analyses for the nine traits mentioned above, which enabled us to classify 300 upland cottons into five clusters, i.e., C1, C2, C3, C4, and C5. We further calculated the composite scores of the five groups through principal component analysis, with higher scores indicating better overall performance of the group. The results showed that the PCA composite scores of C1 and C3 were significantly higher than those of the other groups, but compared with C3, C1 had the highest PCA composite score, compact architecture, smaller FBA, fewer empty fruit branches, more BPPs, and higher yield. Therefore, C1 had the best overall performance, mainly from the NIR, and the FBA of this population ranged between 50.46 and 55.31°. It can be further exploited as an excellent cotton germplasm. This result provides excellent germplasm resources for selection of cotton suitable for different values, which can be used in future selection efforts to provide excellent germplasm resources for breeding.

## 4. Materials and Methods

### 4.1. Plant Materials

In this experiment, we used 300 upland cottons from the National Germplasm Resource Bank, which are widely distributed, including 99 from the YRR, 42 from the YZRR, 89 from the NIR, 43 from the LSMR, and 27 from FG.

### 4.2. Field Trial

The experimental sites were located in different experimental sites in Kuqa City, Xinjiang, planted in the KC1 and KC2 experimental sites in 2022, and in the KC1, KC2, and KC3 experimental sites in 2023 and 2024. The fertility of each experimental site was uniform, and a randomized block design was adopted. Each film covered six rows, with two rows planted for one variety. The row spacing was configured as (66 + 10) cm, and plant spacing of 7 cm was used. Mechanical film-laying and perforation, manual film spot sowing, and sub-film drip irrigation cultivation were employed. The field management methods at each site were the same as conventional field production.

### 4.3. Phenotypic Data Measurement

The FBA of cotton is the angle between the main stem and the fruit branch. At the boll setting stage, when the FBA in cotton had stabilized, we selected six cotton plants with consistent and continuous growth to measure their traits. The average of the six plants was used to represent the phenotypic data of each variety. Previous studies have shown that the upper-middle fruit branch best represents the FBA of the upland cotton as a whole plant and most affects the ventilation and light transmission of the plant [3]. Therefore, we chose to investigate the angle between the middle and upper fruit branches in cotton, i.e., the 4th, 5th, 6th, and 7th-stage fruit branches and the main stem. Measurements were made using an apparent angle ruler. The criteria for investigation of other architecture traits and yield traits are shown in Table 9.

### 4.4. Statistical and Analytical Processing of Data

We used SPSS 27.0 software for descriptive statistical analysis, principal component analysis (no rotation), and significant difference analysis for architecture traits and yield traits. R-4.4.3 software was used for cluster analysis based on the sum of squares of deviations method (Ward.D2) for the FBA in cotton and similarity matrix construction based on Euclidean distance for the plant architecture and yield traits. Origin 2022 software was used to generate frequency distribution histograms and line graphs to visually assess the data distribution.

## 5. Conclusions

In this study, we investigated the multi-year multi-point FBA trait in 300 upland cottons, which demonstrated that the variation of FBA in cotton ranged between 43.59 and 69.32°, the CV ranged between 6.06 and 7.42%, and the broad-sense heritability was 75.50%. These findings indicate that FBA has a wide and stable variation, which is mainly controlled by genetic factors. It is particularly suitable for genetic analyses through QTL mapping and genome-wide association study to identify candidate genes for FBA in cotton.

The cotton FBA in different regions of China showed significant regional differences, having rich genetic diversity. Over time, the FBA in cotton showed a fluctuating decrease, with increasing coefficients of variation and genetic diversity indices. These findings show the direction of regionalized breeding and artificial selection breeding.

This can be used as a core breeding target when the FBA in cotton ranges from 50.46 to 55.31°. At this time, the cotton architecture is compact, with fewer empty fruit branches, more bolls, and higher yields, which meets the requirements of machine picking and production. Therefore, in the future breeding process, breeders should focus on expanding the genetic diversity of the varieties they breed, and on the basis of in-depth analysis of the existing germplasm resources, they should actively explore and make use of foreign germplasm resources and genetic variation in local varieties in China.

## Figures and Tables

**Table 1:** Descriptive statistical analysis of FBA in 2022–2024.

Year	Min	Max	Mean	SD	CV	Skewness	Kurtosis	*H* ^2^
2022	44.57	69.32	55.46 b	4.11	7.42	0.41	0.54	75.50
2023	46.23	66.07	57.68 a	3.50	6.06	−0.07	−0.13	
2024	43.59	63.54	54.41 c	3.77	6.93	0.06	−0.13	
Total mean	47.60	65.16	55.84 b	2.97	5.31	0.17	0.25	

Total mean is the average of each species over a three-year period. Different lowercase letters in the same column represent significant differences between years (*p* < 0.05).

**Table 2:** Variation analysis of the FBA in cotton in the different regions.

Region	No. of Line	Min	Max	Mean	SD	CV	*H*′
YRR	99	49.92	63.32	56.24 ab	3.01	5.35	2.04
LSMR	43	48.14	59.65	55.35 b	2.30	4.15	1.99
NIR	89	48.51	65.27	55.25 b	3.32	6.02	1.94
YZRR	42	49.2	61.38	56.16 ab	2.84	5.06	1.91
FG	27	48.43	61.41	56.77 a	2.94	5.18	1.93

Different lowercase letters in the same column represent significant differences between regions (*p* < 0.05).

**Table 3:** Variation analysis of FBA in cotton during different periods.

Period	No. of Line	Min	Max	Mean	SD	CV	*H*′
S1	19	53.43	61.61	57.28 a	2.06	3.60	1.79
S2	10	54.90	60.07	56.55 a	1.41	2.49	1.42
S3	14	48.43	60.20	54.64 ab	3.19	5.84	1.97
S4	15	50.00	60.77	55.77 ab	2.89	5.19	1.84
S5	34	50.27	59.07	54.59 b	2.23	4.09	2.04
S6	65	48.51	65.27	55.48 ab	3.05	5.50	2.00
S7	70	51.13	63.32	56.80 a	2.99	5.27	2.03
S8	20	48.14	62.44	55.21 ab	3.99	7.22	1.94

S1: Before 1960, S2: 1960s, S3: 1970s, S4: 1980s, S5: 1990s, S6: 2000s, S7: 2010s, S8: 2020s. Different lowercase letters in the same column represent significant differences between periods (*p* < 0.05).

**Table 4:** Variation analysis of different FBA in different periods.

FBA	S1	S2	S3	S4	S5	S6	S7	S8	Range	Mean	SD	CV
FBA4	57.60	56.24	54.95	56.48	55.37	55.97	57.01	56.51	2.65	56.27	0.85	1.51
FBA5	57.23	56.93	54.98	56.12	54.44	55.68	56.90	55.74	2.79	56.00	0.99	1.77
FBA6	57.05	56.57	54.33	55.10	54.37	55.34	56.76	54.21	2.84	55.47	1.17	2.11
FBA7	57.13	56.43	54.25	55.38	54.06	54.91	56.60	54.11	3.07	55.36	1.22	2.21

FBA4, FBA5, FBA6, and FBA7 are the FBA of the 4th, 5th, 6th, and 7th−stage fruit branch in cotton, respectively.

**Table 5:** Variance analysis of six architecture traits and three yield traits in cotton.

Trait	Min	Max	Mean	SD	CV	Skewness	Kurtosis
PH	53.38	98.47	72.60	6.91	9.52	0.57	0.98
FBIN	4.52	8.31	6.05	0.57	9.48	0.44	1.37
FBIH	19.52	42.64	29.24	4.30	14.70	0.45	0.03
FBN	7.29	11.17	8.71	0.63	7.25	0.56	0.97
EFBN	3.98	7.75	5.59	0.60	10.80	0.05	0.07
FBA	47.60	65.16	55.84	2.97	5.31	0.17	0.25
BPP	5.65	15.45	8.50	1.49	17.55	1.22	2.89
BW	4.79	7.95	6.06	0.45	7.34	0.18	1.03
LP	0.31	0.48	0.41	0.03	6.37	−0.70	0.79

**Table 6:** Correlation analysis between architecture traits and yield traits in cotton.

	PH	FBIN	FBIH	FBN	EFBN	FBA	BPP	BW	LP
PH	1								
FBIN	0.335 **	1							
FBIH	0.771 **	0.672 **	1						
FBN	0.060	−0.434 **	−0.370 **	1					
EFBN	0.201 **	−0.115 *	−0.019	0.329 **	1				
FBA	−0.014	0.046	−0.048	−0.039	0.200 **	1			
BPP	0.123 *	−0.085	−0.086	0.360 **	0.675 **	0.080	1		
BW	0.024	0.041	−0.035	−0.044	−0.011	0.131 *	0.044	1	
LP	0.183 **	0.084	0.182 **	−0.186 **	0.127 *	0.110	0.058	−0.090	1

** Correlations are significant at the 0.01 level (two-tailed). * Significant at the 0.05 level (two-tailed).

**Table 7:** Principal component analysis for six architecture traits and three yield traits in cotton.

Trait	Eigenvector			
	Principal Component No. 1	Principal Component No. 2	Principal Component No. 3	Principal Component No. 4
PH	0.62	0.56	−0.27	0.18
FBIN	0.80	0.06	0.10	0.14
FBIH	0.91	0.27	−0.16	0.10
FBN	−0.61	0.41	−0.35	0.19
EFBN	−0.25	0.84	0.08	−0.07
FBA	−0.02	0.23	0.78	−0.10
BPP	−0.31	0.79	0.02	0.05
BW	0.00	0.02	0.54	0.68
LP	0.28	0.26	0.23	−0.71

**Table 8:** Phenotypic analysis of architecture traits and yield traits in five groups in cotton.

Group	No.	PH	FFNP	FFNH	FBN	EFBN	FBA	BPP	BW	LP	PCA Score
C1	9	87.98 a	5.96 b	36.04 a	9.41 a	6.06 a	52.58 c	9.56 a	6.01 ba	0.43 a	0.71 a
C2	24	67.21 d	5.31 c	23.89 d	9.47 a	5.80 a	54.58 b	8.74 b	5.73 c	0.39 bc	−0.82 d
C3	35	77.80 b	6.90 a	35.52 a	8.17 c	5.14 b	54.74 b	7.75 c	5.83 bc	0.42 ab	0.51 ab
C4	114	68.06 d	5.87 b	26.65 c	8.68 b	5.30 b	55.93 ba	7.88 c	6.14 a	0.41 ac	−0.34 c
C5	118	75.13 c	6.12 b	30.46 b	8.69 b	5.92 a	56.75 a	9.20 ab	6.13 a	0.41 ac	0.29 b

Different lowercase letters in the same column represent significant differences between groups (*p* < 0.05).

**Table 9:** Criteria for investigation of other architecture traits and yield traits.

	Trait	Criteria for Investigation
Architecture	PH	Distance from the cotyledonary node of cotton to the tip of the main stem
	FBIN	Node at which the first fruit branch appears on the main stem of cotton
	FBIH	Height from the ground to the first fruit branch
	FBN	FBN per cotton plant
	EFBN	FBN with cotton bolls
Yield	BPP	Number of effective bolls of cotton per plant
	BW	At harvest, 10 middle bolls were collected from each plot, dried, weighed, and divided by 10 to obtain the BW
	LP	At harvest, 10 central bolls were collected from each plot, and the lint weight was divided by the seed cotton weight to give the LP in percent

## Data Availability

The data provided in this study are available upon request from the corresponding author. Due to privacy concerns, these data are not publicly available.

## Abbreviations

The following abbreviations are used in this manuscript:

PCA	Principal Component Analysis
CV	Coefficients of Variation
FG	Foreign Germplasm
YRR	Yellow River Region
YZRR	Yangtze River Region
LSMR	Liaoning Special Maturing Region
NIR	Northwest Inland Region
PH	Plant Height
FBIN	Fruit Branch Initiation Node
FBIH	Fruit Branch Initiation Height
FBN	Number of Fruit Branches
EFBN	Number of Effective Fruit Branches
FBA	Fruit Branch Angle
BPP	Bell Per Plant
BW	Bell Weight
LP	Lint Percentage
*H*′	Genetic diversity index
*H*^2^	Broad-sense heritability

## References

[1] Wei S., Wu J., Zhang S., Liu S. (2012). Advances in Crop Architecture-related Gene Research. Ningxia J. Agri. Fores. Sci. Technol..

[2] Wang Y., Li J. (2008). Molecular Basis of Plant Architecture. Annu. Rev. Plant Biol..

[3] Ma Q., Li J., Xu S., Chen H., Liu W., Ning X., Lin H. (2022). Genetic Analysis of FBA Trait in Upland Cotton with Major Gene Plus Polygenes Mixed Genetic Model. Biotechnol. Bull..

[4] Wang H., Liu C., Tang L., Zhang S., Cai X., Li X., Ma W., Han J., Zhang X., Zhang J. (2023). The influence of different planting densities on agronomic traits, yield and quality of machine—Picked cotton varieties. Xinjiang Agric. Sci..

[5] Yang Y. (2023). Effects of Drip Application of Tyramine and Nitrogen on Cotton Growth and Yield. Master’s Thesis.

[6] He Z., Xi H., Yang B., Li P., Han B. (1984). The Key to Get Good Yield of Cotton by Inducing the Response to Dpc Towards a Planned Direction and in Planned Strength. J. Beijing Univ. Agric..

[7] Meng Y. (2023). Effects of Water, Nitrogen and Film Mulching Mode on the Physiological Characteristics and Yield of Cotton. Master’s Thesis.

[8] Wang S., Pei D., Jia J., Wang H., Wang Z., Zhang X. (2005). The Effects of Irrigation Quantity and Frequency of Drip Irrigation to Plastic-mulched Cotton at Critical Growing Stage. Acta Agric. Boreali Sin..

[9] Xue J., Qi B., Ma B., Li B., Gou L. (2021). Effect of Altered Leaf Angle on Maize Stalk Lodging Resistance. Crop Sci..

[10] Wang B., Wu Y., Huang N., Zhu X., Guo W., Zhang T. (2006). QTL Mapping for Plant Architecture Traits in Upland Cotton Using RILs and SSR Markers. J. Genet. Genom..

[11] Su J., Li L., Zhang C., Wang C., Gu L., Wang H., Wei H., Liu Q., Huang L., Yu S. (2018). Genome-Wide Association Study Identified Genetic Variations and Candidate Genes for Plant Architecture Component Traits in Chinese Upland Cotton. Theor. Appl. Genet..

[12] Liu Q., Wang Y., Zhang X., Wang Q., Li C. (2021). Relationship Between Plant Type Traits and Lint Yield in Upland Cotton in Different Ecological Environments. Southwest China J. Agric. Sci..

[13] He Q. (2018). Functional Characterization of Panicle Architecture Gene Osafb6 and Tillering Angle Gene Lazyi in Rice. Ph.D. Thesis.

[14] Godbolé R., Michalke W., Nick P., Hertel R. (2000). Cytoskeletal Drugs and Gravity-Induced Lateral Auxin Transport in Rice Coleoptiles. Plant Biol..

[15] Yu B., Lin Z., Li H., Li X., Li J., Wang Y., Zhang X., Zhu Z., Zhai W., Wang X. (2007). TAC1, a Major Quantitative Trait Locus Controlling Tiller Angle in Rice. Plant J. Cell Mol. Biol..

[16] Dong H., Zhao H., Xie W., Han Z., Li G., Yao W., Bai X., Hu Y., Guo Z., Lu K. (2016). A Novel Tiller Angle Gene, TAC3, Together with TAC1 and D2 Largely Determine the Natural Variation of Tiller Angle in Rice Cultivars. PLoS Genet..

[17] Li H., Sun H., Jiang J., Sun X., Tan L., Sun C. (2021). TAC4 Controls Tiller Angle by Regulating the Endogenous Auxin Content and Distribution in Rice. Plant Biotechnol. J..

[18] Wang W., Gao H., Liang Y., Li J., Wang Y. (2022). Molecular Basis Underlying Rice Tiller Angle: Current Progress and Future Perspectives. Mol. Plant.

[19] Cai Y., Xiao N., Chen Z., Wu Y., Yu L., Liu J., Shi W., Fan C., Li Y., Zhou C. (2023). Research Progress on Molecular Mechanisms Regulating Rice Tiller Angle. J. Plant Genet. Resour..

[20] Araniti F., Talarico E., Madeo M.L., Greco E., Minervino M., Álvarez-Rodríguez S., Muto A., Ferrari M., Chiappetta A., Bruno L. (2023). Short-Term Exposition to Acute Cadmium Toxicity Induces the Loss of Root Gravitropic Stimuli Perception through PIN2-Mediated Auxin Redistribution in *Arabidopsis thaliana* (L.) Heynh. Plant Sci..

[21] Nan D., Zhao H., Wu Y., Qin C., Nie A., Yang S., Fan Z., Chen Q. (1995). Mechanism of Increasing Yield and Its Technical Studies for Cotton Plant Pattern Cultivation. Cotton Sci..

[22] Ma X., Zhao S., Dong C., Zhou X., Wang X., Li B. (2018). Correlation Analyses between the Main Plant Ideotype Traits and the Yield of Early—Maturing Upland Cotton. Xinjiang Agric. Sci..

[23] Zhu S., Li X. (1980). Cotton architecture breeding. Cotton.

[24] Tang S., Xiao Y., Yang W. (2006). The Analysis of Raw Cotton Fiber Quality between Region and Year in China. Chin. Agric. Sci. Bull..

[25] Pan X., Sun Z., Feng Y., Liu Q., Zhang F. (2011). Growth Stage and Growth Analysis on Cotton with Different Accumulate Temperature in North of Xinjiang. Chin. Agric. Sci. Bull..

[26] Bao Y., Liu A., Chen Q., Li C., Liu Z., Zhang D., Xu J. (2014). Development trend and variety choice of mechanical harvest of precocious cotton in the north of Xinjiang. China Cotton.

[27] Li L., Chang H., Zhao S., Liu R., Yan M., Li F., El-Sheery N.I., Feng Z., Yu S. (2024). Combining High-Throughput Deep Learning Phenotyping and GWAS to Reveal Genetic Variants of Fruit Branch Angle in Upland Cotton. Ind. Crops Prod..

[28] Doust A.N. (2007). Grass Architecture: Genetic and Environmental Control of Branching. Curr. Opin. Plant Biol..

[29] Yang X., Su J., Wu Y., Zhang F., Guan Z., Chen F., Fang W. (2018). Mixed inheritance analysis of branching traits in spray cut chrysanthemum. J. Nanjing Agric. Univ..

[30] Shao P., Peng Y., Wu Y., Wang J., Pan Z., Yang Y., Aini N., Guo C., Shui G., Chao L. (2022). Genome-Wide Association Study and Transcriptome Analysis Reveal Key Genes Controlling Fruit Branch Angle in Cotton. Front. Plant Sci..

[31] Xiong W., Xiong Y. (2024). Studies on the Influence of Meteorological Factors on the Occurrence Pattern of Crop Pests and Diseases. J. Agric. Catastrophology.

[32] Wang Y., Wang S., Zhang Q., Feng G., Lei X., Liang Q., Qi H. (2019). Correlation Analysis between Main Agronomic Traits and Density in Mechanical Harvest Cotton. Crops.

[33] Yan P. (2020). Effects of Density on Boll-Forming Spatial and Temporal Distribution and Yield Formation of Short-Growth-Duration Direct Seeding Cotton. Master’s Thesis.

[34] Wang B., Smith S.M., Li J. (2018). Genetic Regulation of Shoot Architecture. Annu. Rev. Plant Biol..

[35] Zhou X., Fu C., Ma C., Wang X., Zhao C. (2021). Research Progress of Molecular Regulation of Branching of Crops. Biotechnol. Bull..

[36] Kuai J., Sun Y., Zuo Q., Huang H., Liao Q., Wu C., Lu J., Wu J., Zhou G. (2015). The Yield of Mechanically Harvested Rapeseed (*Brassica napus* L.) Can Be Increased by Optimum Plant Density and Row Spacing. Sci. Rep..

[37] Chen M., Yang Y., Wang Y., Tian J., Xu S., Liu N., Dang K., Zhang W. (2019). Plant Type Characteristics and Evolution of Main Economic Characters in Early Maturing Upland Cotton Cultivar Replacement in Xinjiang. Sci. Agric. Sin..

[38] Wang Y., Zhang Q., Dong M., Li B., Wang S., Feng G., Liang Q., Qi H. (2023). Effects of row spacing and density on plant architecture, yield and fiber quality of machine-picked cotton in southern Hebei cotton region. J. Nanjing Agric. Univ..

[39] Fu Y., Li P., Wang H., Wang Q., Li C. (2016). Relationship of Plant Architecture Traits and Lint Yield of Upland Cotton Variety (Line) Resources. Southwest China J. Agric. Sci..

